# Refractory Cryptoglandular Perianal Abscess and Fistulas Due to Mycobacterium avium Infection in an Immunocompetent Adult

**DOI:** 10.7759/cureus.67827

**Published:** 2024-08-26

**Authors:** Hannah Y Lyons, Jackson R Brunner, Markos Mardourian, Johan Nordenstam, Gautam S Kalyatanda

**Affiliations:** 1 Division of Infectious Diseases and Global Medicine, College of Medicine, University of Florida, Gainesville, USA; 2 Department of Surgery, University of Florida, Gainesville, USA

**Keywords:** nontuberculous mycobacterium (ntm), enteric fistula, mycobacterium avium, immunocompetent patient, perianal abscess

## Abstract

*Mycobacterium avium* is a slow-growing nontuberculous mycobacterium (NTM) requiring prolonged treatment with multiple antimicrobials. It primarily affects immunocompromised patients and causes infection of the respiratory tract, skin, and soft tissue. While enteric carriage of *M. avium* has been reported, it has not been associated with clinical infection in immunocompetent hosts. To our knowledge, this is the first case report of a perirectal abscess caused by primary *M. avium* infection in an otherwise healthy patient and indicates the importance of considering NTMs as causative organisms in intraabdominal and enteric abscesses even among immunocompetent individuals when multiple courses of antibiotics are ineffective.

## Introduction

*Mycobacterium avium* is a slow-growing nontuberculous mycobacterium (NTM) that primarily affects patients with immunocompromising conditions such as human immunodeficiency virus (HIV) infection, treatment with immunomodulatory biological agents, and transplant-associated immunosuppression [1,2]. NTMs are aerobic, acid-fast organisms that are generally considered to have a high antimicrobial resistance due to cell wall impermeability, biofilm formation, and their ability to survive in warm temperatures [3]. NTM infections often result in nonspecific patient presentations, and organism identification often requires molecular testing of specimens. Additionally, slow-growing species take up to six weeks to mature colonies and require specialized growth media and staining methods, making culture-based data difficult to apply in clinical scenarios [4].

*M. avium* most commonly causes pulmonary disease in patients with underlying lung disease, although cases of *M. avium* disseminated disease, lymphadenitis, skin and soft-tissue infection, and tenosynovitis have been reported [5]. NTMs are ubiquitous in the environment, and inhalation and ingestion are considered the leading routes of transmission for pulmonary *M. avium* infection [3,6]. While enteric carriage of *M. avium* has been reported, it is not commonly associated with clinical infection in immunocompetent hosts with nondisseminated disease. Skin and soft-tissue NTM infections are relatively uncommon but should be considered if a superficial infection does not resolve with an initial course of antibiotics [7]. Disseminated NTM infections may present with scattered skin lesions, but this is rarely the primary site of infection. Skin and soft-tissue NTM infections typically present as sequelae of trauma, surgery, or cosmetic procedures. There are no unifying radiographic findings of extra-pulmonary *M. avium* infection, and imaging is not required for diagnosis unless there is concern for spread into deeper tissue or adjacent structures [8].

Treatment centers on a multidisciplinary approach, often necessitating antimicrobial susceptibility testing and prolonged multidrug therapy [9,10]. Bronchial hygiene and clearance regimens as well as surgical resection may serve as adjuvant therapies to reduce bacterial burden [3]. The standard antimicrobial treatment regimen includes combination therapy with a macrolide, a rifamycin, and ethambutol. Amikacin is occasionally added as a fourth agent [5]. Macrolides and amikacin are the only agents that have demonstrated clinical responses to *in-vitro* susceptibility patterns, so antibiotic susceptibility testing for wider panels of antimicrobials is generally recommended only in cases of macrolide resistance [4]. Investigations are currently underway to establish the *in vivo* efficacy of agents like clofazimine, bedaquiline, and tedizolid in *M. avium* infection. Duration of treatment is based on clinical judgment rather than evidence-based guidelines and is often abbreviated due to treatment intolerance or drug-related adverse events [4,11]. Treatment with three agents is typically required for 6-12 months [12].

## Case presentation

**Figure 1:**
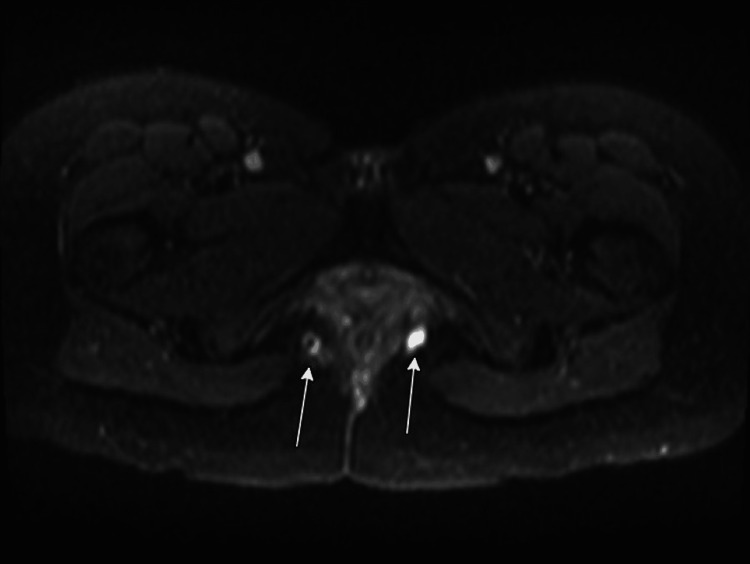
MRI demonstrating complex fistula (yellow) with bilateral (L > R), evolving ischiorectal abscesses (white). During the patient’s first procedure at our institution, surgeons located the internal opening of the abscess in the posterior midline, just distal to the dentate line. It was found to have extension from the posterior midline to both the left and right.

**Figure 2:**
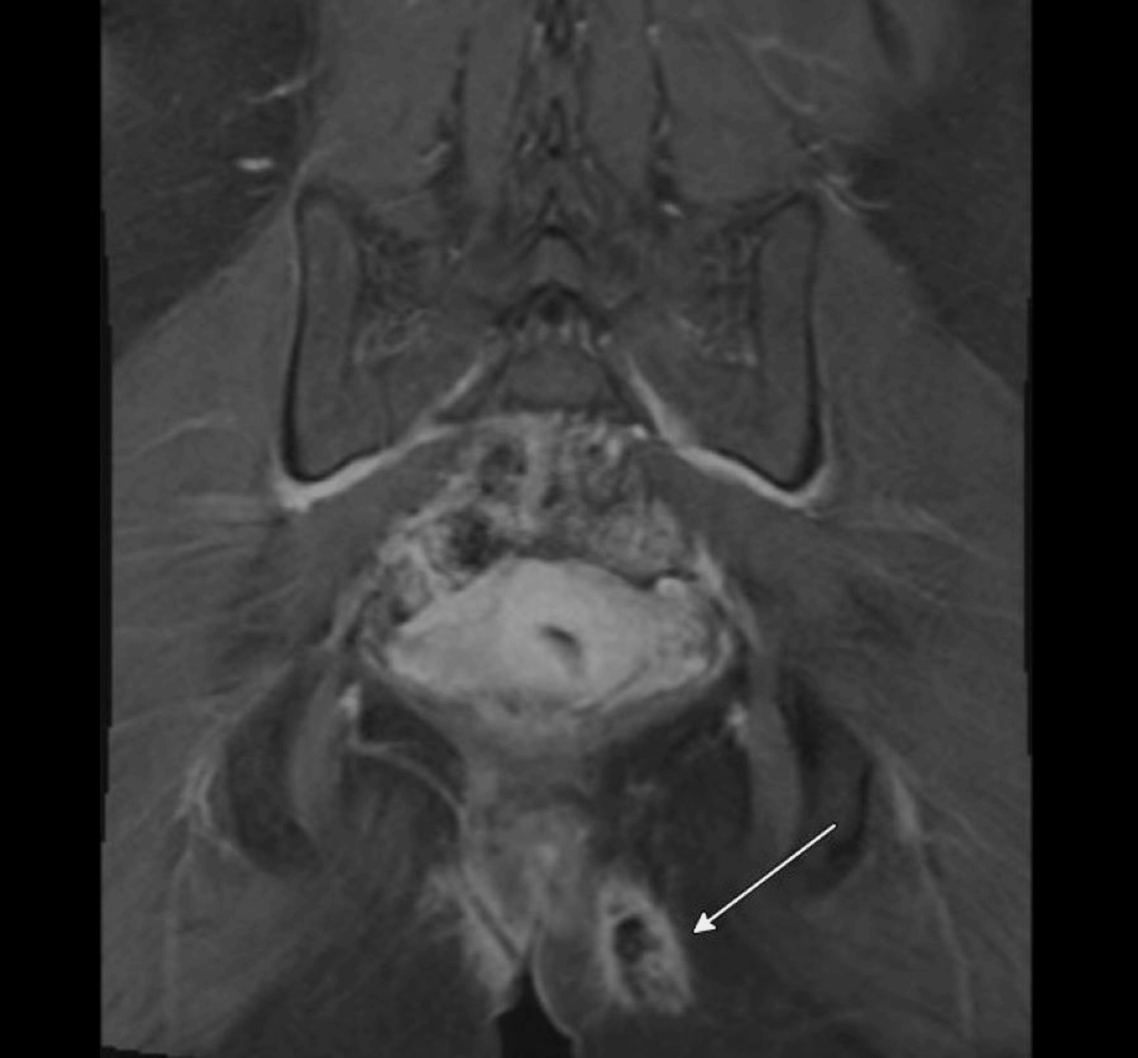
Coronal view of patient’s fistula tract (white) which communicates with the anterior perianal region.

A 37-year-old female with no significant past medical history presented to our infectious disease clinic for evaluation of a chronic painful perianal fistula and abscess with associated discharge. The patient’s symptoms first began six months prior to presentation after she underwent a Brazilian wax and developed a swelling in her left gluteal region. At an outside institution, a computed tomography (CT) scan was performed, which did not reveal Fournier’s gangrene or abscess formation, leading to a diagnosis of cellulitis. Despite a 10-day course of oral amoxicillin/clavulanic acid followed by a five-day course of oral trimethoprim-sulfamethoxazole (TMP-SMX), the cellulitis progressed, and she developed an abscess. An incision and drainage (I&D) was performed, but unfortunately, no intraoperative cultures were sent. Over a span of two months, she was administered empiric azithromycin, amoxicillin/clavulanic acid, and ciprofloxacin due to persistent symptoms. Subsequent magnetic resonance imaging (MRI) confirmed the presence of a complex fistulous tract suggesting bilateral ischiorectal space abscesses and a horseshoe fistula (Figures 1, 2). She was then referred to the colorectal surgery department at our institution due to persistence of the abscess.

At our institution, she underwent three procedures: 1) I&D and bilateral seton placement from abscess internal opening to its bilateral extensions, 2) bilateral anal fistulectomy, and 3) I&D of ischiorectal space abscesses over a seven-month period. During this time, she also received empiric ciprofloxacin and metronidazole for three weeks without any improvement of her symptoms. One month after the third procedure, she presented to the emergency department with left-sided anal pain, associated with fever and chills. Labs were notable for a white blood cell count of 8.8k/cm^3^ with a normal differential, erythrocyte sedimentation rate of 39 mm/hr, C-reactive protein of 71.93 mg/L, and lactate of 0.8 mmol/L. On physical examination, the left gluteus was indurated and there was an unusual milk-colored drainage from the left anal wound. She was started on IV vancomycin and piperacillin/tazobactam, and a CT scan of the abdomen and pelvis revealed a complex anal fistula with an abscess measuring 3.8 cm on the left side. She was taken to the operating room for exam under anesthesia (EUA), I&D of the left-sided perianal abscess, and bilateral seton placement using a modified Hanley procedure. Intraoperatively, a white-colored discharge was noted which was sent for bacterial, fungal, and acid-fast bacteria (AFB) cultures. She was discharged the following day on a three-day course of oral amoxicillin/clavulanic acid.

Blood cultures demonstrated no growth, but operative cultures grew 1+ microaerophilic Streptococcus species, few Staphylococcus aureus, and Escherichia coli. AFB culture grew acid-fast bacilli, which speciated to *M. avium*, susceptible to amikacin and clarithromycin, at which point the patient was referred to our infectious disease clinic for further management. She was started on daily oral azithromycin 500mg PO, daily oral rifampin 600 mg, daily oral ethambutol 800 mg, and amikacin 25 mg/kg via a peripherally inserted central catheter three times weekly. In addition to the above antimicrobials, she completed a seven-day course of ciprofloxacin 500mg PO BID to cover the other organisms isolated from cultures. Her symptoms, including the discharge, improved soon after the three-drug regimen was initiated. Amikacin was discontinued after two months, and MRI three months after initiation of therapy revealed a persistent transsphincteric fistula tract consistent with previous seton placement and abscess resolution. Eight months after initiation of therapy, the patient underwent repeat EUA, fistula debridement, and seton exchange with intraoperative AFB cultures showing no growth. She then underwent fistula repair and advancement flap closure with complete resolution of drainage, bleeding, and pain. Rifampin, ethambutol, and azithromycin were discontinued after eight months of therapy, and the wound closed with no further treatment or complications.

## Discussion

Gastrointestinal carriage of NTMs is thought to be associated with clinical disease in immunocompromised individuals, causing vague symptoms such as abdominal pain, chronic diarrhea, fever, weight loss, and ascites [13,14]. Pulmonary and disseminated NTM infection is much more common than gastrointestinal infection. In a study of immunocompromised renal transplant patients, 40% of NTM-infected patients presented with disseminated NTMs while only 1.7% of NTM-infected patients had an isolated, nonmetastatic gastrointestinal site of infection [15]. NTM abdominal wall and hepatic abscesses have been reported among patients with HIV and patients treated with monoclonal antibodies or tumor necrosis factor inhibitors, but only as manifestations of disseminated disease rather than the initial foci of infection [16-19]. Thus, it is likely that our colleagues at the initial outside institution did not send AFB cultures given the low pre-test probability for NTM infection in this immunocompetent patient.

Sample collection in cases of enteric infections is often challenging due to the abscess location. Laparoscopy and laparotomy are the best methods to obtain biopsies to identify causative pathogens and ensure appropriate histopathological examination. Culture, followed by PCR speciation and antibiotic susceptibility testing, is the definitive method of identifying causative NTM organisms. Our patient’s abscess cultures demonstrated polymicrobial infection, which reinforces the importance of culture data in appropriately tailoring antimicrobial therapy, as well as the importance of including AFB cultures for the identification of rare causative organisms in nonhealing abdominal wounds. 

While our patient’s symptoms resolved and negative AFB surgical cultures were obtained after eight months of therapy, there is no well-defined treatment endpoint for skin and soft-tissue NTM infections. Superficial NTM infections often necessitate 4-12 months of treatment, but the treatment duration is dependent on clinical improvement and drug-related adverse reactions [20]. This case presents a unique case of NTM infection in an otherwise healthy patient and highlights the importance of future studies to standardize and refine treatment regimens for cutaneous and enteric NTM infections.

## Conclusions

This case illustrates the importance of considering atypical pathogens in perianal abscess cases that are refractory to initial antimicrobial therapy, even when the patient is otherwise healthy and immunocompetent. Cultures and antimicrobial susceptibility profiles are essential to the diagnosis and treatment of superficial infections, and in our case coordination of medical and surgical treatment strategies contributed to a positive patient outcome.

While enteric and cutaneous NTMs in immunocompetent individuals are rare, this case presents a viable eight-month antimicrobial regimen that effectively led to cure. Negative post-therapy surgical cultures were considered an adequate treatment endpoint in this case, but such cultures would likely not be feasible to collect in patients with less extensive surgical reconstruction. For this reason, our case also highlights the lack of consensus regarding treatment duration and endpoints for cutaneous and enteric NTM infection, and additional investigation is needed to better standardize treatment strategies. 
